# Association of uric acid with kidney function and albuminuria: the Uric Acid Right for heArt Health (URRAH) Project

**DOI:** 10.1007/s40620-021-00985-4

**Published:** 2021-03-23

**Authors:** Elisa Russo, Francesca Viazzi, Roberto Pontremoli, Carlo Maria Barbagallo, Michele Bombelli, Edoardo Casiglia, Arrigo Francesco Giuseppe Cicero, Massimo Cirillo, Pietro Cirillo, Giovambattista Desideri, Lanfranco D’Elia, Claudio Ferri, Ferruccio Galletti, Loreto Gesualdo, Cristina Giannattasio, Guido Iaccarino, Giovanna Leoncini, Francesca Mallamaci, Alessandro Maloberti, Stefano Masi, Alessandro Mengozzi, Alberto Mazza, Maria Lorenza Muiesan, Pietro Nazzaro, Paolo Palatini, Gianfranco Parati, Marcello Rattazzi, Giulia Rivasi, Massimo Salvetti, Valérie Tikhonoff, Giuliano Tocci, Andrea Ungar, Paolo Verdecchia, Agostino Virdis, Massimo Volpe, Guido Grassi, Claudio Borghi, on behalf of the Working Group on UricAcid and Cardiovascular Risk of the Italian Society of Hypertension

**Affiliations:** 1 Department of Internal Medicine, University of Genoa and IRCCS Ospdedale Policlinico San Martino, Viale Benedetto XV, 6, 16132 Genoa, Italy; 2 Biomedical Department of Internal Medicine and Specialistics, University of Palermo, Palermo, Italy; 3 Department of Medicine and Surgery, Clinica Medica, University of Milano-Bicocca, Monza, Italy; 4 Department of Medicine, Studium Patavinum, University of Padua, Padua, Italy; 5 Department of Medical and Surgical Science, Alma Mater Studiorum University of Bologna, Bologna, Italy; 6 Department of Public Health, Federico II University of Naples Medical School, Naples, Italy; 7 Department of Emergency and Organ Transplantation-Nephrology, Dialysis and Transplantation Unit, Aldo Moro University of Bari, Bari, Italy; 8 Department of Life, Health and Environmental Sciences, University of L’Aquila, L’Aquila, Italy; 9 Department of Clinical Medicine and Surgery, Federico II University of Naples Medical School, Naples, Italy; 10 Cardiology IV, A. De Gasperis Department, Health Science Department, Niguarda Ca’ Granda Hospital, Milano-Bicocca University, Milan, Italy; 11 Department of Advanced Biomedical Sciences, Federico II University of Naples Medical School, Naples, Italy; 12 Reggio Cal Unit, CNR-IFC, Clinical Epidemiology of Renal Diseases and Hypertension, Reggio Calabria, Italy; 13 Department of Clinical and Experimental Medicine, University of Pisa, Pisa, Italy; 14 Department of Internal Medicine, Hypertension Unit, General Hospital, Rovigo, Italy; 15 Department of Clinical and Experimental Sciences, University of Brescia, Brescia, Italy; 16 Department of Medical Basic Sciences, Neurosciences and Sense Organs, University of Bari Medical School, Bari, Italy; 17 S. Luca Hospital, Istituto Auxologico Italiano & University of Milan-Bicocca, Milan, Italy; 18 Department of Medicine, Medicina Interna 1°, Ca’ Foncello University Hospital, University of Padova, Treviso, Italy; 19 Department of Medicine, University of Padua, Padua, Italy; 20 Hypertension Unit, Division of Cardiology, Department of Clinical and Molecular Medicine, Faculty of Medicine and Psychology, Sant’Andrea Hospital, University of Rome Sapienza, Rome, Italy; 21 IRCCS Neuromed, Pozzilli, Italy; 22 Department of Geriatric and Intensive Care Medicine, Careggi Hospital and University of Florence, Florence, Italy; 23 Hospital S. Maria della Misericordia, Perugia, Italy

**Keywords:** Serum uric acid, eGFR, Albuminuria, Cardiovascular risk

## Abstract

**Background:**

Hyperuricemia is commonly observed in patients with chronic kidney disease (CKD). However, a better understanding of the relationship among uric acid (UA) values, glomerular filtration rate (GFR) and albuminuria may shed light on the mechanisms underlying the excess of cardiovascular mortality associated with both chronic kidney disease and hyperuricemia and lead to better risk stratification. Our main goal was to study the relationships between serum uric acid and kidney disease measures (namely estimated GFR [eGFR] and albuminuria) in a large cohort of individuals at cardiovascular risk from the URic acid Right for heArt Health (URRAH) Project database.

**Methods:**

Clinical data of 26,971 individuals were analyzed. Factors associated with the presence of hyperuricemia defined on the basis of previously determined URRAH cutoffs for cardiovascular and all-cause mortality were evaluated through multivariate analysis. Chronic kidney disease was defined as eGFR < 60 ml/min per 1.73 m^2^ and/or abnormal urinary albumin excretion diagnosed as: (i) microalbuminuria if urinary albumin concentration was > 30 and ≤ 300 mg/L, or if urinary albumin-to-creatinine ratio (ACR) was > 3.4 mg/mmol and ≤ 34 mg/mmol; (ii) macroalbuminuria if urinary albumin concentration was > 300 mg/L, or if ACR was > 34 mg/mmol.

**Results:**

Mean age was 58 ± 15 years (51% males, 62% with hypertension and 12% with diabetes), mean eGFR was 81 ml/min per 1.73m2^2^with a prevalence of eGFR < 60 and micro- or macroalbuminuria of 16, 15 and 4%, respectively. Serum uric acid showed a trend towards higher values along with decreasing renal function. Both the prevalence of gout and the frequency of allopurinol use increased significantly with the reduction of eGFR and the increase in albuminuria. Hyperuricemia was independently related to male gender, eGFR strata, and signs of insulin resistance such as body mass index (BMI) and triglycerides.

**Conclusions:**

The lower the eGFR the higher the prevalence of hyperuricemia and gout. In subjects with eGFR < 60 ml/min the occurrence of hyperuricemia is about 10 times higher than in those with eGFR > 90 ml/min. The percentage of individuals treated with allopurinol was below 2% when GFR was above 60 ml/min, it increased to 20% in the presence of CKD 3b and rose further to 35% in individuals with macroalbuminuria.

**Graphic abstract:**

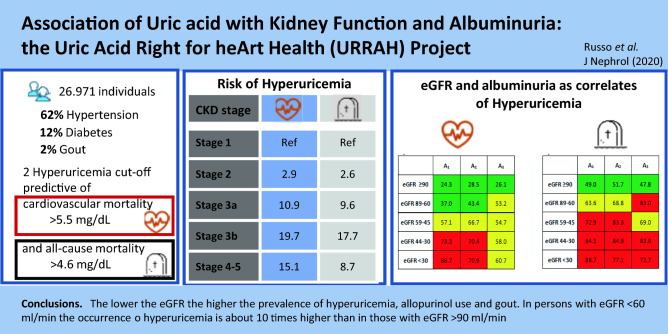

**Supplementary Information:**

The online version contains supplementary material available at 10.1007/s40620-021-00985-4.

## Introduction

Hyperuricemia is an established risk predictor for cardiovascular and kidney diseases [1–4] in populations with [5] and without chronic kidney disease (CKD) [6] independently of the presence of gout. Furthermore, high levels of serum urate, even within the normal range, have been shown to predict the development of albuminuria [7] and both the occurrence and progression of CKD [8–10].

Chronic kidney disease is also a well-known CV risk multiplier on its own. Both reduced glomerular filtration rate (GFR) and albuminuria independently entail an increased risk of mortality, especially from causes [11]. The presence of CKD brings about several changes in renal handling of uric acid (UA), including reduced glomerular filtration, enhanced reabsorption and/or insufficient secretion by renal tubules. These abnormalities account for up to 90% of the mechanisms leading to hyperuricemia and gout [12, 13]. Thus, CKD and hyperuricemia often coexist and serum urate levels increase linearly with decreasing GFR [14].

Therefore, it is not clear whether the increased risk associated with serum uric acid is at least in part related to the concomitant impaired kidney function. A better understanding of the relationship among serum uric acid values, GFR and albuminuria may shed light on the mechanisms underlying the excess of cardiovascular mortality associated with both CKD and hyperuricemia and lead to better risk stratification.

We investigated the relationship between serum uric acid levels and CKD components in the Uric Acid Right for heArt Health (URRAH) Project study population.

## Methods

### Population

The URRAH project is based on a multicenter, retrospective, observational cohort study, which involves data collected on a regional community basis from the whole Italian territory under the patronage of the Italian Society of Hypertension (SIIA). Participating centers which collected the data included in the general database are listed under Acknowledgments. The design and main results of the URRAH study have previously been described [15].

The URic acid Right for heArt Health study was performed according to the Declaration of Helsinki for Human Research (41st World Medical Assembly, 1990). The processing of the individuals’ personal data collected in this study comply with the European Directive on the Privacy of Data. All data collected, stored and processed have been anonymized, and all study-related documents are retained in a secure location. No personal information is stored on local personal computers. Approval was sought from the Ethics Committee of the coordinating center at the Division of Internal Medicine of the University of Bologna (no. 77/2018/Oss/AOUBo). Informed consent was obtained from all individuals at recruitment.

### Data collection

Data concerning individuals (3689; 1910 men, 1779 women) in whom the estimation of eGFR was not possible or for whom data regarding the use of allopurinol were not available were excluded from analysis. Data obtained from the remaining 26,971 individuals (13,768 men, 13,203 women) were included in the present study.

Kidney function was assessed by serum creatinine and urinary albumin excretion measurements. GFR was estimated for each person using a standardized serum creatinine assay and the Chronic Kidney Disease Epidemiology Collaboration formula (eGFR) [16]. Urine samples for albumin excretion measurements were collected before each study visit (usually within one week). Abnormal urinary albumin excretion was diagnosed as: i. microalbuminuria if urinary albumin concentration was > 30 and ≤ 300 mg/L, or if urinary albumin excretion rate was > 20 and ≤ 200 μg/min, or if urinary albumin-to-creatinine ratio (ACR) was > 3.4 mg/mmol and ≤ 34 mg/mmol in both genders; ii. macroalbuminuria if urinary albumin concentration was > 300 mg/L, or if urinary albumin excretion rate was > 200 μg/min, or if ACR was > 34 mg/mmol in both genders. Albuminuria indicates individuals with either micro or macroalbuminuria. Chronic kidney disease was defined as estimated GFR (eGFR) < 60 ml/min per 1.73 m^2^ and/or albuminuria. Hyperuricemia was defined on the basis of the classic cutoff (i.e., ≥ 7 mg/dl) and of the previously described [2] URRAH cutoffs predictive for cardiovascular mortality (i.e. ≥ 5.6 mg/dl) and all-cause mortality (i.e. ≥ 4.7 mg/dl). The diagnosis of gout was attributed on (an history basis). Hypertension was defined according to ESH-ESC guidelines as blood pressure (BP) at least 140/90 mmHg or by the presence of antihypertensive treatment. Systolic and diastolic BP was measured twice, in a quiet room, after 5 min resting and with the participant in the sitting position. The second measurement was used for all analyses.

The main analysis was aimed at evaluating the association among hyperuricemia, the use of allopurinol and kidney disease measures.

### Statistical analyses

The patients’ baseline clinical and demographic characteristics are reported overall, per eGFR strata and per severity of albuminuria as mean and SD for continuous variables normally distributed and as median values (interquartile ranges) for skewed variables. Logarithmically transformed values of skewed variables were used for the statistical analysis. Comparisons between groups were made by analysis of variance. Comparisons of proportions among groups were made using the χ^2^ test. Missing values, when present, were below 5%.

Univariate and multivariate logistic regression analyses were used to describe the relationship between all available clinical variables of biological relevance and the presence of hyperuricemia. Odds ratios and 95% confidence intervals were calculated by exponentiation of logistic regression coefficients.

Statistical calculations were performed by STATA package, version 14.2 (StataCorp, College Station, TX). The null hypothesis was rejected for values of *p* less than 0.05.

## Results

### Clinical characteristics on the basis of eGFR

**Fig. 1:**
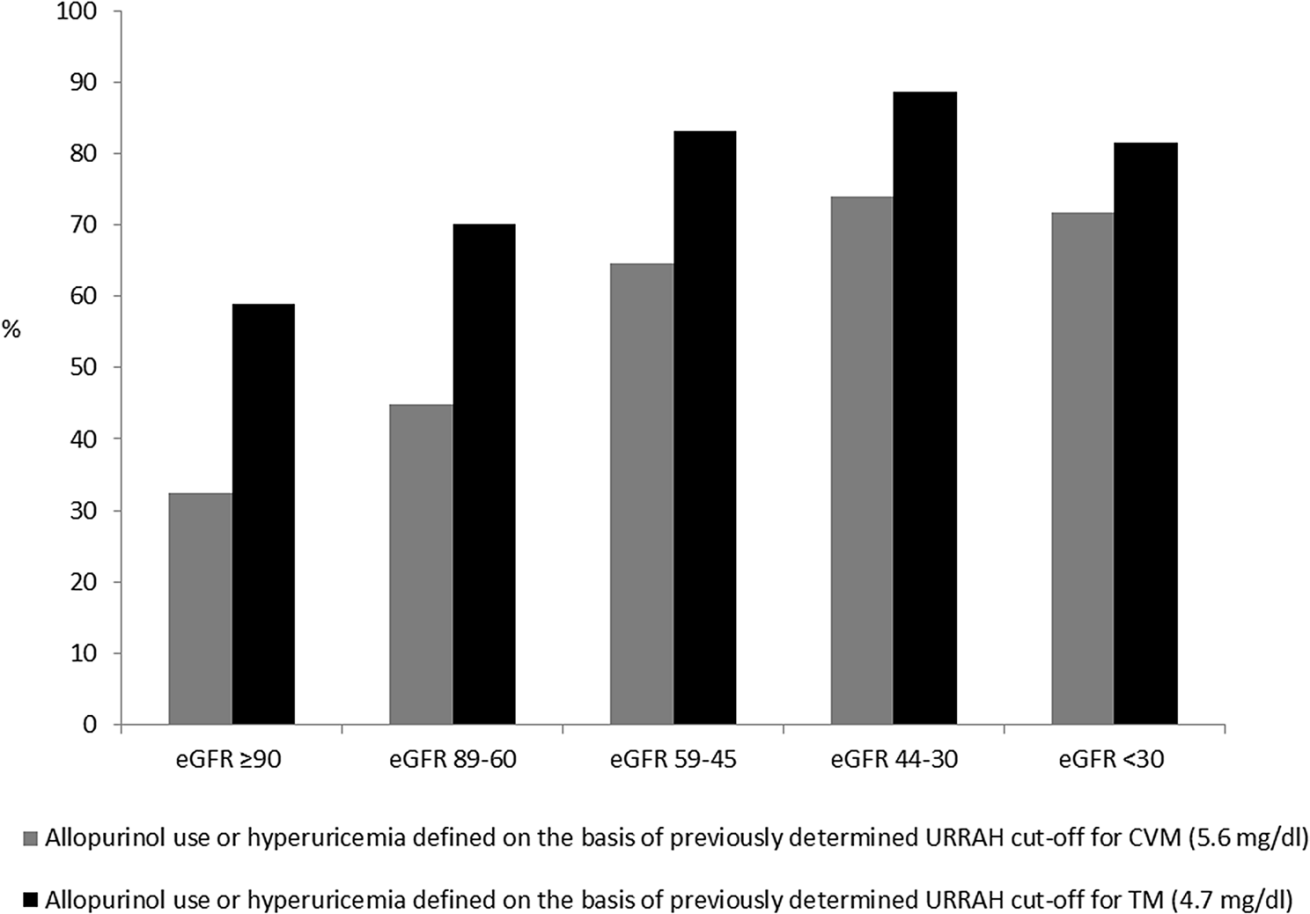
Prevalence of allopurinol use or hyperuricemia on the basis of glomerular filtration rate strata in the whole study population. eGFR, estimated glomerular filtration rate (ml/min per 1.73m^2^); CVM, cardiovascular mortality; ACM, all-cause mortality

The main clinical characteristics of the study population as a whole and when analyzed on the basis of GFR strata are shown in Table 1. Altogether, out of 30,660 individuals, 26,971 for whom complete data on both serum uric acid and eGFR were available were included in the analysis. Mean age was 58 ± 15 years, mean serum uric acid was 5.08 ± 1.44 mg/dl and mean eGFR was 81 ± 21 ml/min per 1.73 m^2^, 51.1% were males, 61.8% had a history of hypertension and 12.3% of diabetes. The overall prevalence of reduced eGFR, (< 60 ml/min per 1.73 m^2^) was 16% and was significantly higher in females as compared to males (23 vs 9% p < 0.0001, data not shown). Data reported in Table 1 show some relevant differences among eGFR subgroups. In fact, individuals with reduced eGFR were more likely to be women and older and with a history of diabetes when compared with those with preserved eGFR. Moreover, they were more likely to be (treated with each class of) anti-hypertensive drugs and statins, with somehow better BP values and lipid profile. As expected, serum uric acid levels and proportion of gout rose along with the increase in eGFR strata, and the prevalence of hyperuricemia (defined on the basis of both the CVM and the ACM thresholds) increased progressively as eGFR decreased, going from 20 and 43% in individuals with an eGFR of at least 90 ml/min per 1.73 m^2^ to 33% and 60% in those with eGFR between 60 and 90 ml/min per 1.73 m^2^, 50 and 75% in those with eGFR between 45 and 60 ml/min per 1.73 m^2^, 59 and 82% in those with eGFR between 30 and 45 ml/min per 1.73m^2^ and 54 and 70% in those with eGFR below 30 ml/min per 1.73m^2^ (p < 0.0001), with a very steep increase in the proportion of allopurinol use from 1, to 2, 7, 21 and 35%, respectively. The sum of these data is depicted in Fig. 1 showing how the prevalence of hyperuricemia or allopurinol use is increasing along with the decrease in GFR. As a matter of fact, the proportion of males with gout being treated with allopurinol is substantially two to three times higher as compared to females in each eGFR strata except for males with eGFR below 30 ml/min who were less frequently treated with allopurinol as compared to females (33 vs 37%, p < 0.001) (data not shown).

**Table 1:** Clinical characteristics on the basis of glomerular filtration rate strata

	All	eGFR ≥ 90 ml/min per 1.73m^2^	eGFR 89–60 ml/min per 1.73m^2^	eGFR 59–45 ml/min per 1.73m^2^	eGFR 44–30 ml/min per 1.73m^2^	eGFR < 30 ml/min per 1.73m^2^	p
N	26,971	9,461	13,222	2,915	954	419	
Age, years	58 ± 15	48 ± 13	61 ± 13	68 ± 12	74 ± 10	73 ± 13	< 0.0001
Males, %	51.1	64.2	48.9	24.4	33.0	49.9	< 0.0001
Smokers, %	24.6	26.5	21.3	19.9	21.1	19.3	< 0.0001
Body mass index, kg/m^2^	27 ± 4	26 ± 5	27 ± 4	27 ± 4	28 ± 4	27 ± 5	< 0.0001
Family history for hypertension, %	53.1	53.8	52.7	53.1	52.6	44.3	0.2686
Diabetes, %	12.3	7.6	12.8	17.7	24.2	39.8	< 0.0001
Hypertension, %	61.8	56.0	64.8	68.2	63.2	53.0	< 0.0001
Systolic BP, mmHg	143.1 ± 22.8	137 ± 21	154 ± 23	150 ± 22	149 ± 24	143 ± 21	< 0.0001
Diastolic BP, mmHg	84.6 ± 12.6	84 ± 12	85 ± 12	85 ± 13	81 ± 14	77 ± 13	< 0.0001
Heart rate, bpm	71 ± 12	72 ± 12	71 ± 11	71 ± 12	71 ± 12	73 ± 13	< 0.0001
Creatinine, mg/dl	0.96 ± 0.37	0.8 ± 0.1	0.9 ± 0.1	1.2 ± 0.1	1.5 ± 0.2	3.0 ± 1.5	< 0.0001
eGFR, ml/min per 1.73m^2^	81 ± 21	103 ± 10	76 ± 8	54 ± 4	39 ± 4	21 ± 7	< 0.0001
Microalbuminuria, %	14.8	11.1	11.3	19.7	42.5	41.2	< 0.0001
Macroalbuminuria, %	4.1	1.0	1.6	5.3	16.4	48.6	< 0.0001
Albuminuria, %	18.9	12.1	12.8	25	59.0	89.7	< 0.0001
Serum uric acid, mg/dl	5.08 ± 1.44	4.61 ± 1.30	5.16 ± 1.36	5.76 ± 1.46	6.15 ± 1.58	5.99 ± 2.22	< 0.0001
Allopurinol use or hyperuricemia ( URRAH cut-off for CVM = 5.6 mg/dl), %	69.1	58.9	70.0	83.1	88.7	81.4	< 0.0001
Allopurinol use or hyperuricemia (URRAH cut-off for ACM = 4.7 mg/dl), %	45.4	32.5	44.9	64.6	73.9	71.7	< 0.0001
Gout, %	1.7	0.8	1.7	2.6	3.3	20.9	< 0.0001
Allopurinol use, %	3.7	1.0	1.7	6.9	21.3	34.7	< 0.0001
Hemoglobin, g/dl	14.2 ± 1.5	14.2 ± 1.4	14.4 ± 1.3	14.2 ± 1.6	13.4 ± 1.7	12.1 ± 1.7	< 0.0001
Glucose, mg/dl	99 ± 25	94 ± 22	100 ± 24	105 ± 30	109 ± 34	108 ± 39	< 0.0001
Cholesterol, mg/dl	209 ± 40	205 ± 39	214 ± 39	210 ± 41	194 ± 44	173 ± 45	< 0.0001
HDL-cholesterol, mg/dl	54 ± 18	55 ± 18	54 ± 18	54 ± 20	53 ± 21	50 ± 18	< 0.0001
LDL-cholesterol, mg/dl	130 ± 38	127 ± 37	134 ± 37	129 ± 39	114 ± 42	94 ± 39	< 0.0001
Triglycerides, mg/dl	128 ± 78	116 ± 78	132 ± 76	142 ± 80	141 ± 75	144 ± 72	< 0.0001
ACE inhibitors,%	16.1	12.2	16.5	24.9	25.5	25.5	< 0.0001
ARB, %	13.2	11.3	12.0	19.4	23.1	20.8	< 0.0001
Calcium channel blockers,%	10.1	8.3	9.7	13.5	18.5	20.5	< 0.0001
Beta blockers, %	9.6	9.5	9.2	11.4	11.3	8.3	0.0032
Diuretics, %	18.8	13.2	17.7	26.5	42.7	66.1	< 0.0001
Statins, %	7.6	5.1	6.7	12	22.3	26.5	< 0.0001

Data presented as mean ± standard deviation or percentage

*eGFR* estimated glomerular filtration rate, *BP* blood pressure, *ACM* all-cause mortality, *CVM* cardiovascular mortality, *SUA* serum uric acid, *HDL* high-density lipoprotein, *LDL* low-density lipoprotein, *ACE* angiotensin converting enzyme, *ARB* angiotensin II receptor blockers

### Clinical characteristics on the basis of albuminuria

**Fig. 2:**
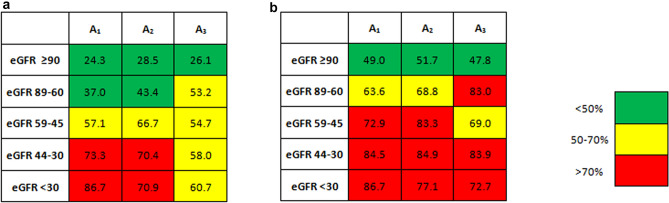
Prevalence of allopurinol use or hyperuricemia accordingly with URRAH cut-offs for **a** cardiovascular and **b** all-cause mortality on the basis of kidney disease measures. eGFR, estimated glomerular filtration rate (ml/min per 1.73m^2^); A_1_, normoalbuminuria; A_2_, microalbuminuria; A_3_, macroalbuminuria

Among 7484 subjects for whom data concerning albuminuria were available, 19% had micro or macroalbuminuria. Individuals with albuminuria were more likely to be males and older and to have eGFR < 60 ml/min and a history of diabetes when compared with those with normoalbuminuria (Table S2). Moreover, they were more likely to be (treated with each class of) anti-hypertensive drugs (especially diuretics) and statins, with somehow better BP values and lipid profile. Serum UA levels and proportion of gout grew along with the severity of albuminuria, and the prevalence of hyperuricemia (both defined on the basis of the cardiovascular and all-cause mortality threshold) increased progressively from 34 and 60% in individuals with normoalbuminuria to 44% and 65% in those with microalbuminuria and 39 and 66% in those with macroalbuminuria (P < 0.001), despite the larger use of allopurinol from 2, to 16 and 35%, respectively. Figure 2 shows how hyperuricemia and the use of allopurinol were more likely associated with eGFR below 45 ml/min and with normo or microalbuminuria rather than with better preserved eGFR or macroalbuminuria.

### Correlates of Hyperuricemia

Individuals with serum uric acid levels over cardiovascular and all-cause mortality threshold were older, had worse renal function and higher albuminuria and showed a positive history for hypertension, diabetes and gout as compared to those with serum uric acid levels below 5.6 mg/dl and 4.7 mg/dl, respectively. In addition, individuals with hyperuricemia were more likely male and smokers and showed higher body mass index (BMI), BP levels, hemoglobin, glucose plasma levels and a worse lipid profile (Table S1). Multiple logistic regression analyses indicate that the main covariates associated with hyperuricemia (both with cardiovascular and all-cause mortality threshold) were CKD stage, male gender, history of hypertension, BMI, and TG levels. In subjects with eGFR < 60 ml/min the occurrence of hyperuricemia was about 10 times higher than in those with eGFR > 90 ml/min. Age was inversely related to the presence of hyperuricemia while history of diabetes lost its statistical significance at multivariate analysis (Table 2). There were 2759 Individuals with serum uric acid levels above 7 mg/dl and 459 with gout.. The risk of having more than 7 mg/dl of serum uric acid or allopurinol prescription or alternatively a positive history for gout increased significantly as eGFR strata decreased, regardless of the presence of diabetes, hypertension or obesity and increased TG levels (Tables S3 and S4).

**Table 2:** Correlates of hyperuricemia defined on the basis of URRAH cutoff predictive for cardiovascular (a) and all-cause (b) mortality

	Univariate				Model 1				Model 2			
	Regr. Coef	OR	CI 95%	p	Regr. Coef	OR	CI 95%	p	Regr. Coef	OR	CI 95%	p
(a)												
eGFR ≥ 90 ml/min per 1.73m^2^		Ref				Ref				Ref		
eGFR 89–60 ml/min per 1.73m^2^	0.691	1.69	1.58–1.81	< 0.0001	1.042	2.84	2.62–3.07	< 0.0001	1.055	2.87	2.62–3.14	< 0.0001
eGFR 59–45 ml/min per 1.73m^2^	1.386	3.80	3.44–4.20	< 0.0001	2.329	10.27	9.08–11.60	< 0.0001	2.394	10.96	9.55–12.56	< 0.0001
eGFR 44–30 ml/min per 1.73m^2^	1.759	5.89	5.02–6.92	< 0.0001	2.806	16.54	13.83–19.79	< 0.0001	2.892	19.73	16.03–24.28	< 0.0001
eGFR< 30 ml/min per 1.73m^2^	1.539	5.25	4.19–6.56	< 0.0001	2.474	11.87	9.33–15.10	< 0.0001	2.716	15.12	11.23–20.37	< 0.0001
Gender, male	0.554	1.74	1.98–2.22	< 0.0001	1.168	3.21	3.01–3.43	< 0.0001	1.214	3.37	3.13–3.62	< 0.0001
Age < 45 years		ref		< 0.0001		Ref				Ref		
Age 45–65 years	0.239	1.27	1.17–1.38	< 0.0001	– 0.173	0.84	0.77–0.92	0.0002	– 0.536	0.58	0.52–0.65	< 0.0001
Age > 65 years	0.145	1.16	1.06–1.26	0.0005	– 0.836	0.43	0.39–0.48	< 0.0001	– 1.343	0.26	0.23–0.29	< 0.0001
Hypertension, presence of	0.497	1.70	1.60–1.81	< 0.0001					0.681	1.98	1.83–2.13	< 0.0001
Diabetes, presence of	0.409	1.31	1.21–1.43	< 0.0001					0.086	1.09	0.98–1.21	0.1069
Body mass index > 30 kg/m^2^	0.503	1.65	1.54–1.78	< 0.0001					0.493	1.64	1.51–1.78	< 0.0001
Triglycerides > 200 mg/dl	0.947	2.58	2.36–2.82	< 0.0001					0.780	2.18	1.97–2.41	< 0.0001
eGFR, each 10 ml/min per 1.73m^2^ increase	– 0.026	0.77	0.76–0.78	< 0.0001								
eGFR < 60 ml/min per 1.73m^2^	1.054	3.10	2.87–3.35	< 0.0001								

	Univariate				Model 1				Model 2			
	Regr. Coef	OR	CI 95%	p	Regr. Coef	OR	CI 95%	p	Regr. Coef	OR	CI 95%	p
(b)												
eGFR ≥ 90 ml/min per 1.73m^2^		ref				ref				ref		
eGFR 89–60 ml/min per 1.73m^2^	0.489	1.63	1.53–1.74	< 0.0001	0.997	2.71	2.51–2.92	< 0.0001	0.974	2.65	2.43–2.88	< 0.0001
eGFR 59–45 ml/min per 1.73m^2^	1.241	3.46	3.09–3.87	< 0.0001	2.192	8.52	7.87–10.18	< 0.0001	2.260	9.58	8.31–11.03	< 0.0001
eGFR 44–30 ml/min per 1.73m^2^	1.699	5.47	4.44–6.74	< 0.0001	2.681	14.61	11.70–18.23	< 0.0001	2.837	17.06	13.23–22.00	< 0.0001
eGFR< 30 ml/min per 1.73m^2^	1.115	3.05	2.37–3.92	< 0.0001	1.865	6.46	4.95–8.43	< 0.0001	2.163	8.70	6.21–12.18	< 0.0001
Gender, male	0.879	2.41	2.27–2.55	< 0.0001	1.260	3.52	3.30–3.76	< 0.0001	1.358	3.89	3.62–4.18	< 0.0001
Age <45 years		ref				ref				ref		
Age 45–65 years	0.262	1.30	1.20–1.41	< 0.0001	– 0.106	0.90	0.82–0.98	0.0176	– 0.438	0.65	0.58–0.72	< 0.0001
Age >65 years	0.113	1.12	1.03–1.21	0.0055	– 0.712	0.49	0.45–0.54	< 0.0001	– 1.217	0.30	0.26–0.33	< 0.0001
Hypertension, presence of	0.565	1.76	1.66–1.87	< 0.0001					0.736	2.08	1.94–2.25	< 0.0001
Diabetes, presence of	0.173	1.19	1.09–1.30	0.0001								
Body mass index > 30 kg/m^2^	0.492	1.63	1.51–1.78	< 0.0001								
Triglycerides > 200 mg/dl	0.882	2.41	2.18–2.68	< 0.0001								
eGFR, each 10 ml/min per 1.73m^2^ increase	– 0.18	0.82	0.81–0.84	< 0.0001								
eGFR < 60 ml/min per 1.73m^2^	1.023	2.78	2.54–3.04	< 0.0001								

*Regr. Coef.* logistic regression coefficient; OR, odds ratio, *CI* confidence intervals, *eGFR* estimated glomerular filtration rate

Among the subjects for whom data about albuminuria were available at univariate analyses, the presence of micro- and macro-albuminuria increased the risk of hyperuricemia defined on the basis of URRAH cutoff predictive for CV mortality by 20 and 30%, respectively (Table S6) and increased the risk of having serum uric acid levels above 7 mg/dl or using allopurinol by three and six times, respectively (Table S8). There was no direct relationship between the presence of albuminuria and the risk of having serum uric acid levels above 4.5 mg/dl (Table S7). Furthermore, there was an inverse relationship between serum uric acid and the presence of macroalbuminuria after adjustment for potential confounding factors (Tables S6 and S7).

## Discussion

In the present cross sectional study we describe the relationship between serum uric acid and kidney disease measures in a very large population of individuals at increased CV risk using data from the URic acid Right for heArt Health (URRAH) study database.

Prevalence of hyperuricemia defined on the basis of previously validated URRAH cutoffs specific for cardiovascular and all-cause mortality was 32 and 57%, respectively, and increased significantly from 20 and 33% in subjects with eGFR > 90 ml/min to 60 and 80% in CKD 3b. As more than 70% of urate is excreted with urine, kidney function impairment is known to cause serum uric acid accumulation [17], thus individuals with CKD are more likely to show hyperuricemia and gout [18] as compared to those with preserved kidney function. Nevertheless, data on the prevalence of hyperuricemia and its relationship with kidney measures have, to date, been limited to small [19] or selected [20] populations. The only previous large study which reports the relationship between gout and eGFR strata relied on administrative codes to define gout [21], and serum uric acid values were not reported. Therefore, our study provides, for the first time to our knowledge, detailed information about the prevalence and correlates of hyperuricemia across different CKD stages.

The overall proportion of individuals with a history of gout was below 2% in our study population. The percentage of individuals treated with allopurinol was below 2% when GFR was above 60 ml/min, it increased to 20% in the presence of CKD 3b and rose further to 35% in individuals with macroalbuminuria (Tables 1 and S5). These data indicate that in real world clinical practice in Italy urate lowering treatment is currently prescribed in a large proportion of individuals with asymptomatic hyperuricemia.

Our data provide new insights into the complex relationship between serum uric acid and CKD. The greater the severity of the CKD stage, the higher the occurrence of hyperuricemia (both by the use of cardiovascular and all-cause mortality threshold) even after adjustment for age, sex, history of hypertension and components of the Metabolic Syndrome such as BMI and TG levels. Individuals with eGFR < 60 ml/min were ten times more likely to have hyperuricemia as compared to those with eGFR > 90 ml/min. From a practical standpoint, physicians should be aware that patients with CKD are at increased risk of presenting hyperuricemia. While the prognostic role of the coexistence of these conditions cannot be investigated in a cross-sectional study, the clustering of both hyperuricemia and CKD with unfavorable clinical and metabolic parameters support the hypothesis of their adverse effects on cardiovascular outcome.

To the best of our knowledge, this is the largest study population to have been investigated with regard to the relationship between serum uric acid levels and the presence of micro and macro-albuminuria. Individuals with hyperuricemia are more likely to have albuminuria (Table S1), and those with albuminuria showed higher serum uric acid levels, more frequent use of allopurinol and history of gout as compared to individuals without albuminuria (Table S5). While the risk of serum uric acid levels above 7 mg/dl or the use of allopurinol is doubled by the presence of albuminuria at multivariate analysis (Table S8), this relationship seems to be largely affected by several confounding factors when a less stringent cutoff is used as is suggested by the inconsistently results of the multiple logistic regression analyses (Tables S6 and S7). Indeed, while male sex, hypertension, diabetes and obesity or insulin resistance are strongly and directly related to both increased serum uric acid levels and albuminuria, these parameters may be influenced by specific conditions in opposite ways. The use of antihypertensive treatment (in particular diuretics) can effectively reduce albuminuria and contribute to a relevant increase in serum uric acid levels, on the other hand, dietary patterns that we did not monitor, such as a high sodium diet, have been shown to be associated with increased albumin excretion rate and might contribute to reducing serum uric acid levels. Moreover, the risk of an over-adjustment must be taken into consideration especially when we included eGFR strata in the models and reduced the sample of the study population to the subgroup of patients with available data on albuminuria. Results of a linear regression analysis between serum uric acid and albuminuria by the 3 different units (mg/dl, mg/die and mg/g) showed, as expected, a direct linear relationship between serum uric acid levels and albuminuria. This relationship reached statistical significance only when albuminuria was measured as mg/dl (p = 0.04) and is largely driven by patients with macroalbuminuria (data not shown). Multiple linear regression analyses confirm the data presented in Tables S6 and S7 indicating that gender, age, diagnosis of hypertension, BMI, triglycerides and eGFR significantly interfere with this relationship (data not shown).

We found that the prevalence of hyperuricemia or allopurinol use was lower in the presence of macroalbuminuria (Fig. 2), especially in individuals with eGFR below 45 ml/min. While this finding is unforeseen, it could be related to a greater prevalence of individuals with diabetes among those with increased albuminuria and decreased eGFR (Tables 1 and S5). In fact, it has been previously reported that in patients with decompensated diabetes, increased glycosuria is accompanied by an increased loss of UA in the urine [22]. The increased traffic of glucose in the tubular lumen causes SGLT2 to be saturated. The attempt to reabsorb the glucose that escaped to the upstream SGLT2 forces the downstream GLUT9 to eliminate more UA in the urine in counter transport with the glucose [23, 24]. This may finally lead to a net negative balance for UA which ultimately results in reduced serum uric acid levels, as seen in clinical practice when SGLT2is are used. Furthermore, it could be hypothesized that albuminuria may signal the severity of tubular damage and therefore might be related to less efficient handling of uric acid along the nephron. However, this hypothesis is not supported by recent preliminary data in CKD patients without diabetes [25].

As a matter of fact, increased serum uric acid levels have been proven to be predictive of kidney disease progression in the early stages of CKD and in individuals without proteinuria rather than in those with more severe kidney damage [26]. A reduction in the serum urate level by allopurinol did not appear to effectively alter the progression of kidney disease in two randomized controlled trials (RCTs) conducted in persons with type 1 diabetes [27] and with stage 3 or 4 CKD [28], respectively. Nevertheless, the demonstration that treatment with xanthine oxidase inhibitors (XOIs) provides effective protection in the subgroup of CKD patients in the early phases of disease but not in more advanced stages [29–33] suggests the usefulness of detailed characterization of individuals who could benefit from urate lowering treatment. It should be noted that the increase in serum uric acid levels associated with eGFR reduction may have, at least in part, different pathogenetic mechanisms depending on whether it occurs in early as opposed to more advanced stages of renal disease. Accordingly, increased xanthine oxidase (XO) activity may be prevalent in the former setting, while reduced excretion of uric acid prevails in the latter. These different mechanisms may potentially explain why drugs that reduce the activity of XO, the key enzyme involved in the production of uric acid, but also a significant source of radical oxygen species, failed to change the progression of kidney diseases when used in stage 3 or 4 CKD and provided renal protection when employed in the initial stages of CKD. Accordingly, the conflicting results reported by previously published studies on the effect of urate lowering treatment on renal outcomes may be due to heterogeneity in the study populations, namely baseline CKD stages, duration of follow-up, definitions of outcomes and concomitant treatments that can affect serum uric acid levels [3]. An interesting hypothesis that has been put forth to account for the apparent discrepancies in reported data, is that the role of serum uric acid might vary on the basis of the degree and severity of the renal disease. Thus, serum uric acid may act as a strong promoter of renal damage and its unfavorable role might be detectable in the early phases of disease while later on it could be diluted with the many other biomarkers typically associated with CKD in its overt phase.

Our report also has some limitations. Among the first, we must acknowledge that laboratory parameters, including serum uric acid, creatinine, and albuminuria were not measured in a single, centralized laboratory and this may have led to some variability in the relationship between serum uric acid and kidney measures. In addition, the URic acid Right for heArt Health (URRAH) study was composed of a population of white ethnicity, which included a percentage of patients selected from Hypertension clinics, thus resulting in a heterogeneous population. The study design is retrospective, and, as such, only associations, but not cause-effect relationships, can be inferred. Moreover, we have no data about urate lowering therapy (ULT) other than Allopurinol.

In conclusion, hyperuricemia with or without urate deposition and the use of allopurinol are frequent findings in CKD. Hyperuricemia appears to be a marker of adverse cardiovascular profile allowing further risk stratification for patients with CKD. The intertwined relationship between hyperuricemia and kidney damage begs the question of whether hyperuricemia and CKD play an independent role in the dramatic increase in cardiovascular and all-cause mortality observed in CKD patients. Furthermore, the very high prevalence of hyperuricemia we found in CKD patients calls for the need to clarify how hyperuricemia should be defined in the presence of CKD and whether urate lowering treatment is useful for cardiovascular and renal protection in individuals with CKD.

## Supplementary Information

Below is the link to the electronic supplementary material.

Supplementary file1 (PDF 406 KB)
