# Supplementary material for: Association of uric acid with kidney function and albuminuria: the Uric Acid Right for heArt Health (URRAH) Project
Source: J Nephrol. 2021 Mar 23;35(1):211–21. doi: 10.1007/s40620-021-00985-4 (PMC8803667; doi:10.1007/s40620-021-00985-4)
Supplement: Supplementary file 1 — Supplementary file1 (PDF 406 KB) [file 40620_2021_985_MOESM1_ESM.pdf]

Supplemental Table 1. Clinical Characteristics on the basis of presence/absence of hyperuricemia accordingly with URRAH cut-offs for cardiovascular (5.6 mg/dl) and all-cause mortality (4.7 mg/dl) or allopurinol use.

	SUA <5.6 mg/dl	SUA ≥5.6 mg/dl	p	SUA <4.7 mg/dl	SUA ≥4.7 mg/dl	p
**N**	9,726	9,821		6,904	15,462	
**Age, years**	60 ±15	61 ±15	0.0001	59 ±15	60 ±15	0.0287
**Males, %**	41.9	60.2	<0.0001	36.3	57.9	<0.0001
**Smokers, %**	20.7	23.4	<0.0001	20.4	23.6	<0.0001
**Alcohol consumption, %**	64.5	65.5	0.1801	62.4	64.6	0.005
**Body mass index, kg/m^2**	26.4 ±4.4	28.0 ±4.2	<0.0001	26.0 ±4.4	27.5 ±4.2	<0.0001
**Family history for hypertension, %**	53.3	52.5	0.3236	53	52.2	0.3163
**Diabetes, %**	12.5	15.8	<0.0001	12.1	14.1	<0.0001
**Hypertension, %**	59.3	62.7	<0.0001	57.4	62.5	<0.0001
**Systolic BP, mmHg**	141.4 ±23.4	145.6 ±22.2	<0.0001	139.9 ±24.0	144.8 ±22.2	<0.0001
**Diastolic BP, mmHg,**	82.5 ±11.7	85.5 ±12.8	<0.0001	81.8 ±11.6	85.3 ±12.5	<0.0001
**Heart rate, bpm**	72 ±12	71 ±12	<0.0001	73 ±12	71 ±12	<0.0001
**Creatinine, mg/dl**	0.89 ±0.34	1.09 ±0.45	<0.0001	0.87 ±0.35	1.03 ±0.41	<0.0001
**eGFR, ml/min per 1.73m^2**	83.6 ±20.0	72.5 ±21.6	<0.0001	85.5 ±20.0	76.1 ±21.0	<0.0001
**eGFR<60 ml/min per 1.73m^2, %**	11	27.7	<0.0001	9.2	21.9	<0.0001
**Normo-micro-macroalbuminuria, %**	77.8-17.2-4.9	74.2-19.7-6.1	0.0062	78.7-16.8-4.6	78.5-16.5-5.0	0.7812
**Serum uric acid, mg/dl**	4.28 ±0.87	6.66 ±1.04	<0.0001	3.79 ±0.67	6.03 ±1.09	<0.0001
**Gout, %**	0.1	4.4	<0.0001	0.1	2.8	<0.0001
**Allopurinol use, %**	0	11.3	<0.0001	0	6.5	<0.0001
**Hemoglobin, g/dl**	14.0 ±1.4	14.5 ±1.6	<0.0001	13.9 ±1.3	14.5 ±1.5	<0.0001
**Glucose, mg/dl**	99 ±28	102 ±24	<0.0001	98 ±29	101 ±24	<0.0001
**Cholesterol, mg/dl**	207 ±40	209 ±41	0.0043	207 ±40	210 ±41	0.0004
**HDL-cholesterol, mg/dl**	56 ±19	51 ±19	<0.0001	57 ±19	52 ±18	<0.0001
**LDL-cholesterol, mg/dl**	129 ±38	128 ±40	0.6699	129 ±37	130 ±39	0.321
**Triglycerides, mg/dl**	117 ±67	152 ±91	<0.0001	112 ±65	141 ±84	<0.0001
**ACE inhibitors,%**	12.5	19	<0.0001	11.1	18	<0.0001
**ARB, %**	10.2	18.4	<0.0001	8.4	16.3	<0.0001
**Calcium channel blockers,%**	7.1	12.6	<0.0001	6.3	11.5	<0.0001
**Beta blockers, %**	13.7	26	<0.0001	12.1	22.3	<0.0001
**Diuretics, %**	6.3	10.7	<0.0001	5.6	9.4	<0.0001

Data presented as mean ± standard deviation or percentage.

Abbreviations: eGFR, estimated glomerular filtration rate; BP, blood pressure; ACM, all-cause mortality; CVM, cardiovascular mortality; SUA, serum uric acid; HDL, high-density lipoprotein; LDL, low-density lipoprotein; ACE, angiotensin converting enzyme; ARB, angiotensin II receptor blockers.

Supplemental Table 2. Correlates of serum uric acid levels at linear regression analyses

	Univariate				Multivariate			
	**Regr. Coef.**	**CI 95%**	**R**	**p**	**Regr. Coef.**	CI 95%	**t-value**	**p**
**Gender, male**	0.38	0.35-0.42	0.133	<0.0001	0.67	0.64-0.71	38.75	<0.0001
**Age, years**	0.01	0.01-0.01	0.135	<0.0001	-0.01	-0.01 -0.01	-16.45	<0.0001
**Body mass index, kg/m^2**	0.08	0.07-0.08	0.237	<0.0001	0.06	0.05-0.06	29.12	<0.0001
**Triglycerides, mg/dl**	0.01	0.01-0.01	0.257	<0.0001	0.00	0.00-0.00	28.54	<0.0001
**eGFR, each 10 ml/min per 1.73m^2 increase**	-0.80	0.80-0.81	0.318	<0.0001	-0.76	0.74-0.76	-56.46	<0.0001

Abbreviations: Regr. Coef., regression coefficient; R, Std coefficient; CI, confidence intervals; eGFR, estimated glomerular filtration rate.

Supplemental Table 3. Correlates of hyperuricemia defined as SUA>7 mg/dl or allopurinol use

	Univariate				Model 1				Model 2			
	Regr. Coef.	OR	CI 95%	p	Regr. Coef.	OR	CI 95%	p	Regr. Coef.	OR	CI 95%	p
**eGFR ≥90 ml/min per 1.73m^2**		ref				ref				Ref		
**eGFR 89-60 ml/min per 1.73m^2**	0.813	2.25	2.03-2.51	<0.0001	0.954	2.59	2.31-2.91	<0.0001	0.939	2.56	2.24-2.92	<0.0001
**eGFR 59-45 ml/min per 1.73m^2**	1.695	5.45	4.80-6.18	<0.0001	2.113	8.28	7.14-9.59	<0.0001	2.106	8.22	6.95-9.71	<0.0001
**eGFR 44-30 ml/min per 1.73m^2**	2.547	12.77	10.90-14.96	<0.0001	2.924	18.62	15.53-22.32	<0.0001	2.949	19.08	15.42-23.61	<0.0001
**eGFR<30 ml/min per 1.73m^2**	3.045	21.01	16.99-25.99	<0.0001	3.288	26.72	21.24-33.62	<0.0001	3.407	30.17	22.76-39.99	<0.0001
**Gender, male**	0.447	1.56	1.45-1.69	<0.0001	0.913	2.49	2.90-2.71	<0.0001	0.956	2.60	2.36-2.87	<0.0001
**Age <45 years**		ref				ref				ref		
**Age 45-65 years**	0.518	1.68	1.48-1.90	<0.0001	0.100	1.10	0.97-1.26	0.1421	-0.066	0.94	0.80-1.09	0.3846
**Age >65 years**	1.043	2.84	2.51-3.20	<0.0001	0.015	1.02	0.88-1.70	0.8301	-0.183	0.83	0.71-0.98	0.0256
**Hypertension, presence of**	0.519	1.68	1.54-1.83	<0.0001					0.494	1.64	1.48-1.82	<0.0001
**Diabetes, presence of**	0.723	2.06	1.87-2.27	<0.0001					0.213	1.24	1.10-1.39	0.0159
**Body mass index >30 kg/m^2**	0.708	2.03	1.86-2.21	<0.0001					0.605	1.83	1.66-2.02	<0.0001
**Triglycerides >200 mg/dl**	1.075	2.93	2.67-3.22	<0.0001					0.918	2.50	2.25-2.79	<0.0001
**eGFR, each 10 ml/min per 1.73m^2 increase**	-0.038	0.68	0.66-0.69	<0.0001								
**eGFR <60 ml/min per 1.73m^2**	1.520	4.57	4.22-4.96	<0.0001								

Abbreviations: Regr. Coef., logistic regression coefficient; OR, odds ratio; CI, confidence intervals; eGFR, estimated glomerular filtration rate.

Supplemental Table 4. Correlates of gout

	Univariate				Model 1				Model 2			
	Regr. Coef.	OR	CI 95%	P	Regr. Coef.	OR	CI 95%	p	Regr. Coef.	OR	CI 95%	p
eGFR ≥90 ml/min per $1.73m^2$		ref				ref				ref		
eGFR 89-60 ml/min per $1.73m^2$	0.737	2.09	1.46-3.00	<0.0001	0.662	1.93	1.30-2.88	0.0010	0.631	1.88	1.22-2.90	<0.0001
eGFR 59-45 ml/min per $1.73m^2$	1.134	3.11	2.00-4.84	<0.0001	1.258	3.52	2.14-5.79	<0.0001	1.166	3.21	1.85-5.57	0.0043
eGFR 44-30 ml/min per $1.73m^2$	1.384	3.99	2.14-7.43	<0.0001	1.499	4.48	2.28-8.78	<0.0001	1.335	3.80	1.78-8.11	0.0006
eGFR<30 ml/min per $1.73m^2$	3.229	30.84	16.96-56.08	<0.0001	3.493	32.89	17.07-63.37	<0.0001	3.531	34.16	16.40-71.17	<0.0001
Gender, male	1.026	2.79	2.12-3.67	<0.0001	1.306	3.69	2.77-4.91	<0.0001	1.428	4.17	3.02-5.76	<0.0001
Age <45 years		ref				ref				ref		
Age 45-65 years	0.558	1.75	0.96-3.18	0.0676	0.328	1.39	0.75-2.57	0.2954	-0.069	0.93	0.49-1.79	0.8361
Age >65 years	1.297	3.66	2.08-6.44	<0.0001	0.746	2.11	1.14-3.89	<0.0001	0.181	1.20	0.61-2.34	0.5969
Hypertension, presence of	1.330	3.81	2.88-5.04	<0.0001					0.616	1.85	1.26-2.72	<0.0017
Diabetes, presence of	0.102	1.11	1.08-1.13	<0.0001					0.896	2.45	1.74-3.36	<0.0001
Body mass index >30 kg/m^2	0.903	2.47	1.90-3.21	<0.0001					0.722	2.06	1.53-2.78	<0.0001
Triglycerides >200 mg/dl	0.705	2.02	1.47-2.77	<0.0001					0.462	1.59	1.12-2.24	0.0087
eGFR, each 10 ml/min per $1.73m^2$ increase	0.921	2.51	1.91-3.30	<0.0001								

Abbreviations: Regr. Coef., logistic regression coefficient; OR, odds ratio; CI, confidence intervals; eGFR, estimated glomerular filtration rate.

Supplemental Table 5. Clinical characteristics on the basis of albumin excretion.

	All	Normoalbuminuria	Microalbuminuria	Macroalbuminuria	p
**N**	7484	6067	1109	308	
**Age, years**	59 **±15**	58 ±14	66 ±14	68 ±14	<0.0001
**Males, %**	54.9	53.5	58.2	70.1	<0.0001
**Smokers, %**	22.0	21.9	22.8	23.1	0.8189
**Alcool consumption, %**	44.6	43.8	51.4	53.8	0.0050
**Body mass index, kg/m^2**	26.6 ±4.3	26.4 ±4.1	27.6 ±4.5	28.0 ±5.6	<0.0001
**Family history for hypertension, %**	62.7	61.9	68.8	65.0	<0.0001
**Diabetes, %**	10.9	6.5	27.5	33.7	<0.0001
**Hypertension, %**	62.3	65.6	61.7	48.1	<0.0001
**Systolic BP, mmHg**	144.7 ±20.4	145.2 ±20.3	142.6 ±20.8	143.5 ±19.9	<0.0001
**Diastolic BP, mmHg,**	84.4 ±13.3	85.2 ±13.0	81.2 ±13.7	78.2 ±12.3	<0.0001
**Heart rate, bpm**	69 ±11	69 ±11	71 ±12	75 ±13	<0.0001
**Creatinine, mg/dl**	1.0 ±0.5	0.9 ±0.2	1.2 ±0.5	2.2 ±1.5	<0.0001
**eGFR, ml/min per 1.73m^2**	78.8 ±23.2	82.6 ±19.8	68.2 ±27.1	43.5 ±28.6	<0.0001
**eGFR<60 ml/min per 1.73m^2**	20.1	13.8	39.7	74.4	<0.0001
**Uric acid, mg/dl**	5.23 ±1.44	5.18 ±1.40	5.45 ±1.56	5.41 ±1.69	<0.0001
**Allopurinol use or hyperuricemia (URRAH cut-off for CVM=5.6 mg/dl), %**	51.7	50.5	54.9	57.1	0.0062
**Allopurinol use or hyperuricemia (URRAH cut-off for ACM=4.7 mg/dl), %**	73.9	73.8	73.6	75.6	0.7812
**Allopurinol use, %**	7.4	2.1	16.5	35.1	<0.0001
**Gout, %**	0.9	0.7	1.7	6.3	<0.0001
**Hemoglobin, g/dl**	14.0 ±1.5	14.3 ±1.3	13.6 ±1.6	12.9 ±1.9	<0.0001
**Glucose, mg/dl**	96 ±21	95 ±18	104 ±30	108 ±39	<0.0001
**Cholesterol, mg/dl**	201 ±40	205 ±38	187 ±42	172 ±45	<0.0001
**HDL-cholesterol, mg/dl**	53 ±15	53 ±15	51 ±15	48 ±14	<0.0001
**LDL-cholesterol, mg/dl**	126 ±37	129 ±35	112 ±38	95 ±40	<0.0001
**Triglycerides, mg/dl**	120 ±70	117 ±67	129 ±85	156 ±85	<0.0001
**ACE inhibitors,%**	19.5	17.0	28.0	31.1	<0.0001
**ARB, %**	15.2	12.2	25.8	25.2	<0.0001
**Calcium channel blockers,%**	10.9	8.7	19.7	21.1	<0.0001
**Beta blockers, %**	10.9	10.5	14.1	5.4	<0.0001
**Diuretics, %**	23.3	16.4	43.4	60.5	<0.0001
**Statins, %**	15.8	12.4	28.3	30.2	<0.0001

Data presented as mean ± standard deviation or percentage.

Abbreviations: eGFR, estimated glomerular filtration rate; BP, blood pressure; ACM, all-cause mortality; CVM, cardiovascular mortality; SUA, serum uric acid; HDL, high-density lipoprotein; LDL, low-density lipoprotein; ACE, angiotensin converting enzyme; ARB, angiotensin II receptor blockers.

Supplemental Table 6. Correlates of hyperuricemia defined on the basis of URRAH cutoff predictive for cardiovascular in patients with available albuminuria (N=7484)

	Univariate				Model 1				Model 2			
	Regr. Coef.	OR	CI 95%	p	Regr. Coef.	OR	CI 95%	p	Regr. Coef.	OR	CI 95%	p
**eGFR ≥90 ml/min per 1.73m^2**		ref				ref				ref		
**eGFR 89-60 ml/min per 1.73m^2**	0.707	2.03	1.79-2.30	<0.0001	1.141	2.65	2.65-3.70	<0.0001	1.148	3.15	2.66-3.73	<0.0001
**eGFR 59-45 ml/min per 1.73m^2**	1.845	6.33	5.14-7.80	<0.0001	2.915	13.86	13.86-24.58	<0.0001	2.967	19.44	14.56-25.94	<0.0001
**eGFR 44-30 ml/min per 1.73m^2**	1.835	6.27	4.81-8.16	<0.0001	3.053	14.49	14.49-30.96	<0.0001	3.205	24.65	16.69-36.40	<0.0001
**eGFR<30 ml/min per 1.73m^2**	1.396	4.04	3.02-5.39	<0.0001	2.317	6.76	6.76-15.21	<0.0001	2.741	15.50	9.81-24.50	<0.0001
**Gender, male**	0.624	1.87	1.68-2.08	<0.0001	0.916	2.50	2.16-2.88	<0.0001	0.943	2.57	2.22-2.98	<0.0001
**Age <45 years**		ref				ref				ref		
**Age 45-65 years**	0.126	1.13	0.96-1.33	0.1272	-0.472	0.62	0.51-0.76	<0.0001	-0.476	0.62	0.51-0.76	<0.0001
**Age >65 years**	0.293	1.34	1.14-1.57	0.0004	-1.128	0.32	0.25-0.41	<0.0001	-1.145	0.32	0.25-0.40	<0.0001
**Hypertension, presence of**	0.172	1.19	1.05-1.34	<0.0001	0.523	1.69	1.44-1.97	<0.0001	0.525	1.69	1.45-1.97	<0.0001
**Diabetes, presence of**	0.144	1.15	0.98-1.35	0.0752	-0.196	0.82	0.65-1.04	0.1040	-0.128	0.88	0.69-1.12	0.3015
**Body mass index >30 kg/m^2**	0.589	1.80	1.56-2.09	<0.0001	0.574	1.78	1.49-2.12	<0.0001	0.581	1.79	1.50-2.13	<0.0001
**Triglycerides >200 mg/dl**	0.951	2.59	2.12-3.15	<0.0001	0.864	2.37	1.87-3.00	<0.0001	0.891	2.44	1.92-3.09	<0.0001
**Albuminuria, absence**		ref								ref		<0.0001
**Microalbuminuria**	0.177	1.19	1.04-1.37	0.0115					-0.064	0.94	0.76-1.15	0.5364
**Macrolbuminuria**	0.266	1.30	1.03-1.65	0.0267					-0.865	0.42	0.28-0.63	<0.0001
**eGFR, each 10 ml/min per 1.73m^2 increase**	-0.027	0.76	0.74-0.78	<0.0001								

Supplemental Table 7. Correlates of hyperuricemia defined on the basis of URRAH cutoff predictive for all-cause mortality in patients with available albuminuria (N=7484)

	Univariate				Model 1				Model 2			
	Regr. Coef.	OR	CI 95%	p	Regr. Coef.	OR	CI 95%	p	Regr. Coef.	OR	CI 95%	p
**eGFR ≥90 ml/min per 1.73m^2**		ref				ref				ref		
**eGFR 89-60 ml/min per 1.73m^2**	0.730	2.07	1.83-2.35	<0.0001	1.281	3.60	3.03-4.29	<0.0001	1.283	3.61	3.03-4.30	<0.0001
**eGFR 59-45 ml/min per 1.73m^2**	1.649	5.20	4.09-6.61	<0.0001	2.953	19.17	13.84-26.54	<0.0001	2.979	19.67	14.20-27.26	<0.0001
**eGFR 44-30 ml/min per 1.73m^2**	1.697	5.46	3.89-7.66	<0.0001	3.097	22.14	13.80-35.52	<0.0001	3.207	24.70	15.31-39.84	<0.0001
**eGFR<30 ml/min per 1.73m^2**	0.757	2.13	1.56-2.91	<0.0001	1.875	6.52	4.18-10.18	<0.0001	2.224	9.24	5.63-15.16	<0.0001
**Gender, male**	0.810	2.25	2.00-2.52	<0.0001	1.199	3.32	2.85-3.85	<0.0001	1.214	3.37	2.90-3.91	<0.0001
**Age <45 years**		ref				ref				ref		
**Age 45-65 years**	0.195	1.21	1.03-1.43	0.0212	-0.365	0.69	0.56-0.85	0.0005	-0.364	0.69	0.57-0.85	0.0005
**Age >65 years**	0.166	1.18	1.00-1.39	0.0483	-1.250	0.29	0.22-0.37	<0.0001	-1.252	0.29	0.22-0.37	<0.0001
**Hypertension, presence of**	0.299	1.35	1.19-1.52	<0.0001	0.626	1.87	1.60-2.19	<0.0001	0.628	1.87	1.60-2.19	<0.0001
**Diabetes, presence of**	-0.033	0.97	0.81-1.15	0.7094	-0.371	0.69	0.53-0.89	0.0048	-0.291	0.75	0.57-0.97	0.0313
**Body mass index >30 kg/m^2**	0.612	1.84	1.55-2.19	<0.0001	0.591	1.81	1.47-2.21	<0.0001	0.600	1.82	1.49-2.23	<0.0001
**Triglycerides >200 mg/dl**	0.795	2.21	1.75-2.81	<0.0001	0.665	1.94	1.46-2.59	<0.0001	0.684	1.98	1.49-2.64	<0.0001
**Albuminuria, absence**		ref								ref		
**Microalbuminuria**	-0.012	0.99	0.85-1.15	0.8728					-0.177	0.84	0.67-1.04	0.1164
**Macrolbuminuria**	0.091	1.09	0.84-1.43	0.5062					-0.701	0.50	0.50-0.76	0.0015
**eGFR, each 10 ml/min per 1.73m^2 increase**	-0.024	0.79	0.77-0.81	<0.0001								

Abbreviations: Regr. Coef., logistic regression coefficient; OR, odds ratio; CI, confidence intervals; eGFR, estimated glomerular filtration rate.

Supplemental Table 8. Correlates of hyperuricemia defined as SUA>7 mg/dl or allopurinol use in patients with available albuminuria (N=7484)

	Univariate				Model 1				Model 2			
	Regr. Coef.	OR	CI 95%	p	Regr. Coef.	OR	CI 95%	p	Regr. Coef.	OR	CI 95%	p
eGFR ≥90 ml/min per $1.73m^2$		ref				ref				ref		
eGFR 89-60 ml/min per $1.73m^2$	0.632	1.88	1.57-2.25	<0.0001	0.833	2.30	1.83-2.89	<0.0001	0.855	2.35	1.87-2.96	<0.0001
eGFR 59-45 ml/min per $1.73m^2$	1.441	4.22	3.40-5.24	<0.0001	1.986	7.29	5.43-9.78	<0.0001	1.920	6.82	5.07-9.17	<0.0001
eGFR 44-30 ml/min per $1.73m^2$	2.474	11.87	9.26-15.22	<0.0001	3.091	22.00	15.35-31.52	<0.0001	2.856	17.40	12.01-25.20	<0.0001
eGFR<30 ml/min per $1.73m^2$	2.702	14.91	11.12-19.98	<0.0001	3.177	23.98	15.87-36.22	<0.0001	2.691	14.75	9.38-23.21	<0.0001
Gender, male	0.431	1.54	1.35-1.75	<0.0001	0.714	2.04	1.71-2.44	<0.0001	0.626	1.87	1.56-2.23	<0.0001
Age <45 years		ref				ref				Ref		
Age 45-65 years	0.347	1.41	1.14-1.76	0.0017	-0.244	0.80	0.612-1.03	0.0869	-0.244	0.78	0.61-1.01	0.0638
Age >65 years	0.872	2.39	1.94-2.95	<0.0001	-0.484	0.62	0.46-0.82	0.0010	-0.535	0.59	0.44-0.78	0.0003
Hypertension, presence of	0.138	1.15	0.99-1.33	<0.0001	0.641	1.90	1.57-2.29	<0.0001	0.654	1.92	1.59-2.33	<0.0001
Diabetes, presence of	0.810	2.25	1.88-2.68	<0.0001	0.398	1.49	1.16-1.91	0.0017	0.194	1.21	0.94-1.57	0.1376
Body mass index, kg/m^2	0.704	2.02	1.73-2.36	<0.0001	0.538	1.71	1.42-2.06	<0.0001	0.527	1.69	1.40-2.04	<0.0001
Triglycerides, mg/dl	1.044	2.84	2.36-3.41	<0.0001	0.830	2.29	1.84-2.85	<0.0001	0.822	2.27	1.82-2.83	<0.0001
Albuminuria, absence		ref								Ref		
Microalbuminuria	1.077	2.94	2.52-3.42	<0.0001					0.734	2.08	1.67-2.59	<0.0001
Macrolbuminuria	1.833	6.25	4.93-7.93	<0.0001					0.768	2.16	1.48-3.14	<0.0001
eGFR, each 10 ml/min per $1.73m^2$ increase	-0.035	0.70	0.69-0.72	<0.0001								

Abbreviations: Regr. Coef., logistic regression coefficient; OR, odds ratio; CI, confidence intervals; eGFR, estimated glomerular filtration rate.
